# Concurrent immune checkpoint blockade for enhanced cancer immunotherapy utilizing engineered hybrid nanovesicles

**DOI:** 10.3389/fphar.2024.1487940

**Published:** 2024-11-11

**Authors:** Yuxuan Liu, Fuxu Yang, Zhimin Li, Ting Wang, Yeteng Mu, Yuxin Fan, Han Xue, Xiuli Hu, Xingang Guan, Hongxia Feng

**Affiliations:** ^1^ Department of Dermatology, The Affiliated Wenling Hospital of Taizhou University, Taizhou, China; ^2^ College of Medical Technology, Beihua University, Jilin, China; ^3^ Medical School, Taizhou University, Taizhou, China; ^4^ Institute of Polymer Science and Engineering, School of Chemical Engineering, Hebei University of Technology, Tianjin, China

**Keywords:** PD-1, SIRPα, cell membrane nanovesicle, immune checkpoint blockade, cancer immunotherapy

## Abstract

Immune checkpoint inhibitors (ICIs) have revolutionized cancer treatment, demonstrating unprecedented efficacy against advanced cancers. However, their clinical applications are significantly hampered by low overall response rates. Dual blockade of two immune checkpoints represents a promising strategy to enhance immunotherapeutic efficacy. In this study, we developed hybrid cell membrane nanovesicles adorned with PD-1 and SIRPα receptors for combination immunotherapy in melanoma. Our hybrid nanovesicles (PD-1/SIRPα NVs) demonstrated high specificity to PD-L1 and CD47 ligands, facilitating the phagocytosis of melanoma cells by macrophages. In a melanoma mouse model, PD-1/SIRPα NVs significantly suppressed 77% of tumor growth and elicited a robust antitumor immune response for immunotherapy. In conclusion, our findings highlight the promising potential of PD-1/SIRPα NVs as novel and effective ICIs for cancer immunotherapy.

## 1 Introduction

Immune checkpoint inhibitors (ICIs), aimed to revive the exhausted immune cells for systemic antitumor immune response, have revolutionized cancer treatment with unprecedented efficacy in treating many advanced cancers (Johnson et al., 2022; Bagchi et al., 2021). Despite their great clinical success, only a few patients benefit from ICIs as a single treatment. Low response, immune-related adverse events (irAEs), and tumor resistance significantly limit the further application of ICIs (Bagchi et al., 2021; de Miguel and Calvo, 2020). Hence, there is an urgent need to develop novel drugs with enhanced efficacy for cancer immunotherapy (Fang W. et al., 2023; Chen et al., 2023).

Combining ICIs targeting two (or more) immune checkpoint receptors represents an effective strategy for improved cancer treatment (Willsmore et al., 2021; Burton and Tawbi, 2021). For example, combination therapy with anti-CTLA-4 plus anti-PD-1/PD-L1 has shown an outperformed therapeutic effect over single ICIs treatment in melanoma and non-small cell lung cancer (NSCLC) (Chae et al., 2018). The FDA recently approved a combination of PD-1 inhibitor (nivolumab) with LAG-3 inhibitor (relatlimab-rmbw) to treat advanced melanoma. PD-1/LAG-3 co-blockade showed prolonged progression-free survival and enhanced objective response rates in cancer patients compared to PD-1 blockade monotherapy (Abi-Aad et al., 2023). Given the superior efficacy of dual immune checkpoint inhibitor blockade, developing smart formulations (et. bispecific antibody, nanoparticle, fusion protein) for killing two birds with one stone holds advantageous potential for potentiated cancer immunotherapy (Wang R. et al., 2023; Gao et al., 2023; Meng et al., 2021).

The significant advance in nanotechnology has opened a new avenue for effective cancer therapy (Fan et al., 2023; Ren et al., 2021). Cell membrane nanovesicles (CMN) have emerged as a promising delivery platform in tumor diagnosis and cancer treatment (Le et al., 2021; Yang et al., 2022; Zhao et al., 2024). Due to the natural cell-derived biomaterial, cell membranes have inherently good biocompatibility and reduced immunogenicity for therapeutic use (Cheng et al., 2023). When transformed into nanovesicles, CMN exhibits good stability and prolonged circulation *in vivo* compared with synthetic nanocarriers (Mu et al., 2023). More importantly, CMN can be engineered to display a variety of functional proteins on the outer surface, conferring nanovesicles targeting delivery, therapeutic potential, or immunomodulatory properties for treating diseases (Fang T. et al., 2023; Yu et al., 2022; Zhang et al., 2023). Furthermore, by incorporating various therapeutic agents into its inner cavity, CMN could be developed into a versatile platform for combination cancer therapy (Hu et al., 2024; Wang M. et al., 2023). Notably, due to the inherent fusion ability of cell membranes, hybrid CMN could be prepared using two or more types of cell membranes for multifunctional application (Rao et al., 2020; Sun et al., 2023; Guo et al., 2024; Peng et al., 2024).

**SCHEME 1:**
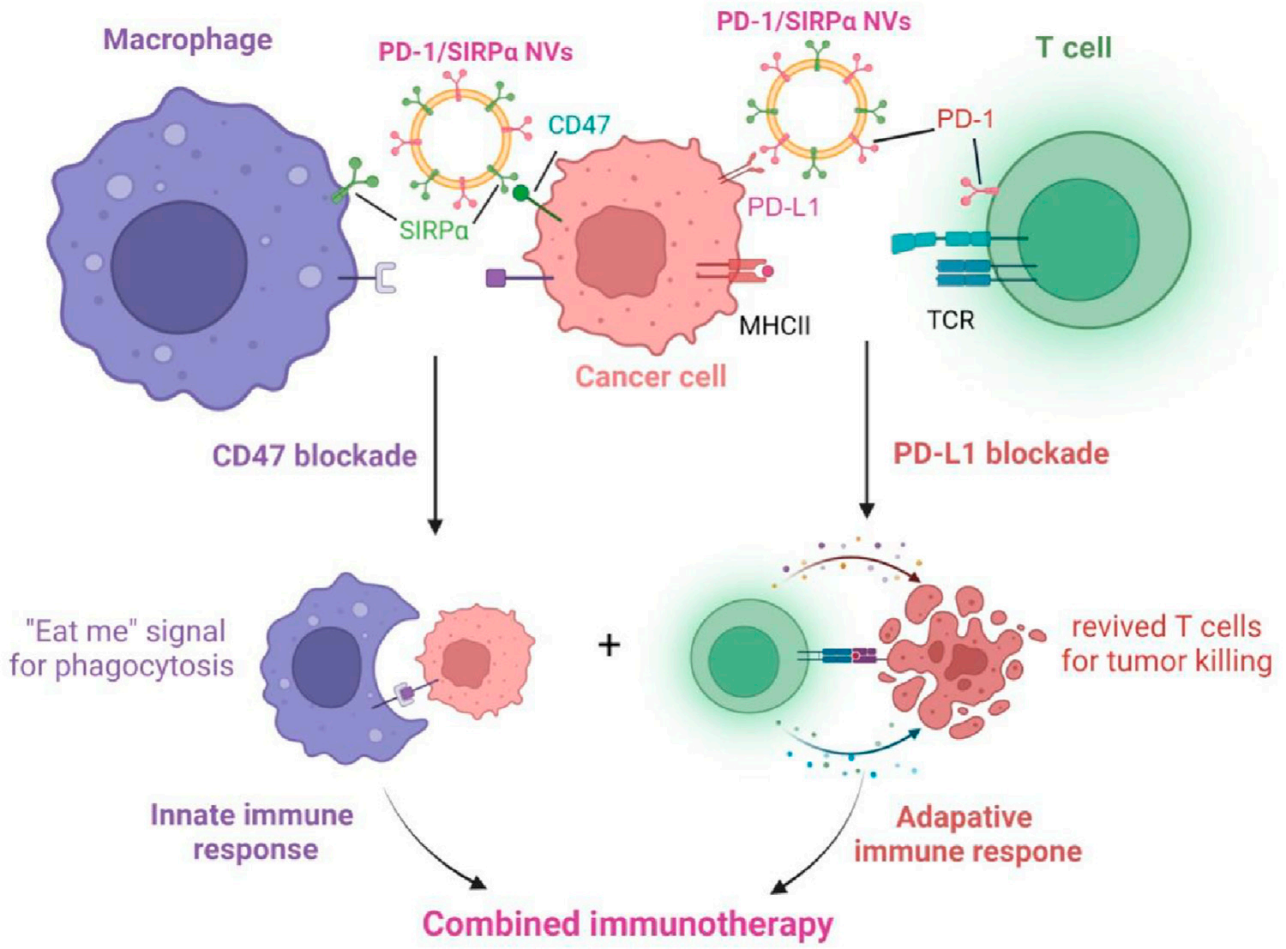
Schematic illustration of the mechanism of PD-1/SIRPα nanovesicles disrupting PD-L1/PD-1 and CD47/SIRPα axes for enhanced cancer immunotherapy.

Herein, we fabricated a hybrid cell membrane nanovesicle with PD-1 and SIRPα co-decoration for combination immunotherapy in melanoma. Given the vital roles of PD-1/PD-L1 and SIRPα/CD47 axes in releasing ‘do not find me’ and ‘do not eat me’ signals (Xiao et al., 2023; Luo J. Q. et al., 2023), we aim to simultaneously disrupt these signals for inducing innate and adaptive immunity using an engineered nanovesicle (Scheme 1). *In vitro* cellular uptake, tumor-selective binding, and *in vivo* antitumor efficacy of PD-1/SIRPα NVs were investigated in detail.

## 2 Materials and methods

### 2.1 Materials

The lentiviral plasmids carrying PD-1-GFP or SIRPα-GFP were purchased from Origen. CellTracker Green and Red were purchased from Thermo Fisher. Antibodies targeting PD-1, SIRPα, and Na^+^K^+^ ATPase for Western blot analysis were purchased from Abcam. All antibodies used for blocking and flow cytometry in this study were obtained from BioLegend. 4′,6-diamidino-2-phenylindole (DAPI), perchlorate (DiO), perchlorate (DiI), and Cell Counting Kit-8 were purchased from Beyotime. Serum tumor necrosis factor-alpha (TNF-α) and interferon-γ (IFN-γ) ELISA kits were purchased from Keygen Biotech.

### 2.2 Cell culture

Murine melanoma cell B16F10, dendritic cells DC2.4, and HEK293T cells were cultured in Dulbecco’s Modified Eagle’s Medium (DMEM) containing 10% Fetal Bovine Serum (FBS) in a cell incubator at 37°C and 5% CO_2_.

### 2.3 Preparation of PD-1 or SIRPα stable cells

Stable cells expressing mouse PD-1 or SIRPα receptors were prepared by transfecting lentiviral vectors carrying PD-1-GFP or SIRPα-GFP into HEK293T cells. Briefly, HEK293T cells were seeded in a six-well plate at a density of 1 × 10^6^ per well. On the second day, Opti-MEM medium containing 6 μL Lipofectamine 3,000 reagent or 4 μg lentiviral plasmids was mixed at room temperature for 15 min and added into cells. After 2 days of transfection, the transfected cells were cultured under the puromycin selection (5 μg/mL). Stable cell lines with PD-1 or SIRPα expression were obtained from single-cell colonies through limited dilution.

### 2.4 Synthesis and characterization of PD-1/SIRPα NVs

HEK293T cells stably expressing SIRPα-GFP and PD-1-GFP were cultured in a DMEM medium containing 10% FBS. The Cells were washed at least three times with cold PBS by centrifuging at 1,000 rpm. Then, the cells were disrupted in a homogenization medium (HM) containing 0.25 M sucrose, 1 mM EDTA, 20 mM Hepes-NaOH, pH 7.4, and a proteinase inhibitor mixture by a Dounce homogenizer for at least 50 times on ice. The supernatant obtained after low-speed centrifugation of entire solutions was ultracentrifuged at 35,000 rpm for 2 h. The pellets were collected and washed with HM buffer containing proteinase inhibitor three times and then resuspended with suitable PBS. After sonicating for 5 min, the cell membranes in PBS finally passed stepwise through 1.0 and 0.4 μm nanopore polycarbonate membranes on an extruder at least 20 times. PD-1 NVs and SIRPα NVs were obtained. As for the hybrid nanovesicle preparation, the PD-1 cell membrane and SIRPα cell membrane were mixed (protein weight ratio of 1:1) and then extruded through 0.4 μm pores on the mini extruder. The obtained nanovesicle was named PD-1/SIRPα NVs. The quantification of nanovesicles was determined by measuring the total protein content within the nanovesicles using a BCA assay. The hydrodynamic diameter and zeta potential of cell membrane nanovesicles were monitored by dynamic light scatter (DLS). The morphologies of NVs were also observed by using transmission electron microscopy (TEM).

### 2.5 Biocompatibility analysis

Five thousand DC2.4 cells were seeded into each well in a 96-well plate and cultured for 12 h. Different concentrations of PD-1/SIRPα NVs were added to the cell medium for 48 h incubation. CCK-8 solution was added to each well for 4 h at 37°C. The absorbance of the solution in each well was determined at 450 nm wavelength using a microplate reader (TECAN M200).

### 2.6 *In vitro* blockade analysis

*In vitro* blockade analysis was performed by assessing the inhibitory binding effect of aPD-L1 on the interaction between nanovesicles and melanoma cells. Dil-labeled PD-1/SIRPα NVs were utilized to track the binding ability. B16F10 cells were seeded in a 12-well plate containing at a density of 2 × 10^5^ cells per well for 12 h. On the second day, the melanoma cells were treated with CD47 antibody solution (50 μg/mL in DMEM), PD-L1 antibody solution (50 μg/mL in DMEM), or CD47 antibody plus PD-L1 antibody solution for 4 h at 37°C. PBS solution was used as a control. The cells were treated with Dil-labeled PD-1/SIRPα NVs for 2 h. After washing with PBS, the melanoma cells were subjected to flow cytometry analysis (gated at PE channel).

### 2.7 Nanovesicles binding assay

B16F10 cells were seeded in a six-well plate and cultured for 12 h. On the second day, mouse red blood cells (RBC) were added to the well. Dil-labeled PD-1/SIRPα NVs (100 μg/mL) were added to the wells containing mixed cells and incubated for 4 h. After that, RBCs were isolated from the supernatant, while B16F10 cells were digested with trypsin and collected. The nanovesicle binding was analyzed by flow cytometry.

### 2.8 *In vitro* phagocytosis assay

Bone marrow-derived macrophages (BMDMs) were isolated from C57BL/6 mice according to the literature. The femur and tibia were taken from mice to acquire bone marrow cells. After spinning at 1,000 rpm for 5 min, the bone marrow cells were mixed with red blood cell lysis buffer at room temperature for 3 min to clear away red blood cells. The marrow cells were washed and placed in DMEM with monocyte colony-stimulating factor (50 ng/mL) for 7 days. Fresh DMEM with M-CSF was changed every 2 days. At the 7-day mark, the sticking cells were colored with CD11b and F4/80 antibodies from Biolegend. The colored cells were confirmed to be BMDMs for later use.

We explored the phagocytosis of tumor cells using BMDMs isolated from C57BL/6 mice. The isolated BMDMs were cultured in DMEM supplemented with 20 ng/mL monocyte colony-stimulating factor (M-CSF) for 7 days to promote differentiation into macrophages. BMDMs were labeled with CellTracker Green, while B16F10 melanoma cells were stained with CellTracker Red. BMDMs were seeded at a density of 2 × 10^5^ cells per well in a six-well plate and cultured overnight. Cells were then exposed to aCD47 or PD-1/SIRPα NVs in a serum-free medium for 2 h. Subsequently, CellTracker Red-stained B16F10 melanoma cells were added, and the mixture was incubated at 37°C for 4 h. After three washes, the uptake of B16F10 cells (labeled red) by BMDMs (labeled green) was visualized using fluorescent microscopy.

### 2.9 *In vivo* antitumor assay

All animal experiments were conducted following the National Institutes of Health Guidelines for the Care and Use of Laboratory Animals, and the guidelines were approved by the Animal Protection and Utilization Committee of Taizhou University (Taizhou, Zhejiang). Five million B16F10 cells were implanted into the right flanks of female C57BL/6 mice to obtain subcutaneous melanoma xenograft. When the tumor size reached 50–80 mm^3^, mice were randomly divided into five groups (n = 5): PBS, Blank NVs (prepared by HEK293T cell membrane without immune checkpoint receptors), PD-1 NVs, SIRPα NVs, and PD-1/SIRPα NVs. The mice were treated with PBS, Blank NVs (25 mg/kg, protein weight), PD-1 NVs (25 mg/kg, protein weight), SIRPα NVs (25 mg/kg, protein weight), and PD-1/SIRPα NVs (25 mg/kg, protein weight) for five times every 3 days via tail vein injection. Tumors volume (V) was calculated to be V = d^2^ × D/2, where d is the shortest, and D is the longest diameter of the tumor, respectively. Animals were euthanized when exhibiting signs of impaired health or when the tumor volume exceeded 2 cm^3^.

### 2.10 Flow cytometry

Immune cells infiltrated in tumor tissues were isolated for flow cytometry. The cell suspension was filtered with a 200 mesh filter and centrifuged at 1,000 rpm for 5 min. The collected cells were treated with the Zombie Violet dye Fixable Viability Kit and blocked with the FC blocking antibody CD16/32 (clone 93). Antibodies targeting CD45 (PerCP-Cyanine5.5; clone 30-F11), CD3 (APC; clone 17A2), CD8a (PE; clone 53–6.7), CD4 (FITC; clone GK1.5), CD80 (PECyanine7; clone 16-10A1), CD86 (APC; clone GL-1), CD206 (APC; clone C068C2) (Biolegend) were used to stain the relative protein. After washing with PBS, the cell suspensions were subjected to a CytoFLEX flow cytometer (Beckman) with CytoExpert software (Beckman Coulter).

### 2.11 Tissue section staining and cytokine detection

Mouse tumors and main organs, including heart, liver, spleen, lung, and kidney, were collected and subjected to hematoxylin and eosin (H&E) staining. Serum samples were isolated from mice treated with different formulations on day 15th after injection. According to the manufacturer’s protocols, the IFN-γ and TNF-α in serum were determined by ELISA (KeyGen).

### 2.12 Statistical analysis

All results are expressed as the mean ± standard deviation. Statistical analysis was performed using SPSS software. One-way or two-way analysis of variance (ANOVA) and Tukey *post hoc* tests were used when more than two groups were compared (multiple comparisons) as indicated. All statistical analyses were carried out using the IBM SPSS statistics 19. The threshold for statistical significance was P < 0.05.

## 3 Results

### 3.1 Preparation and characterization of PD-1/SIRPα NVs

**FIGURE 1:**
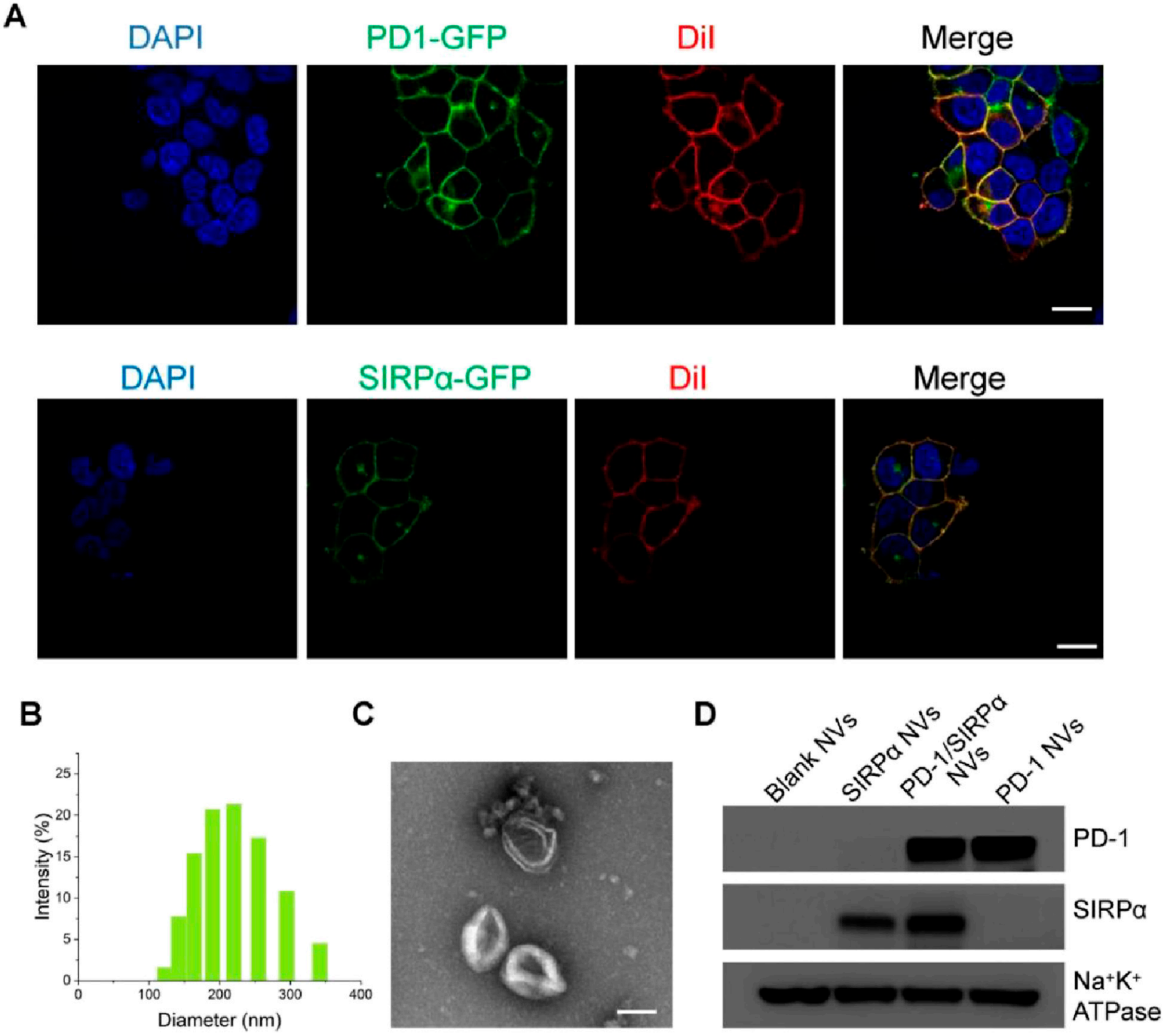
Preparation and characterization of PD-1/SIRPα NVs. **(A)** Confocal microscopy images of PD-1 and SIRPα stable cell lines. Stable cell lines were obtained by transfecting lentiviral vectors carrying PD-1-GFP or SIRPα-GFP through limited dilution. The cell membranes were stained with the membrane dye Dil (red). Scale bar: 10 μm. **(B)** Size distribution of PD-1/SIRPα NVs in PBS solution determined by dynamic light scattering (DLS). **(C)** Morphology analysis of PD-1/SIRPα NVs by TEM. Scale bar: 100 nm. **(D)** Western blot analysis of PD-1 and SIRPα protein expression in hybrid nanovisicles. Na^+^K^+^ ATPase was used as a positive control for membrane protein retained in cell membrane nanovesicles.

To achieve hybrid nanovesicles presenting PD-1 and SIRPα receptors, we generated stable cell lines expressing PD-1-GFP or SIRPα-GFP in HEK293T cells, chosen for their high membrane receptor expression. The stable cell lines were created via transfection with lentiviral plasmids carrying PD-1-GFP or SIRPα-GFP and selection with puromycin using a limited dilution method. Confocal laser scanning microscopy (CLSM) demonstrated the co-localization of PD-1 or SIRPα proteins with the cell membrane probe (Dil), indicating successful membrane expression of the two immune checkpoint receptors (Figure 1A). PD-1 or SIRPα-decorated cell membranes were then isolated via ultracentrifugation and transformed into PD-1 nanovesicles (PD-1 NVs) or SIRPα nanovesicles (SIRPα NVs) by extruding them through 1.0, 0.4, and 0.2 µm pore-sized polycarbonate membrane filters. Finally, PD-1/SIRPα NVs were prepared by extruding mixed cell membranes (protein ratio 1:1) through the mentioned filters. Dynamic light scattering (DLS) analysis revealed that PD-1/SIRPα NVs have a mean diameter of 190.6 nm (Figure 1B). The zeta potential of the hybrid NVs was determined to be −11.5 mV. Morphological analysis via transmission electron microscopy (TEM) indicated that the hybrid nanovesicles possessed a hollow sphere nanostructure (Figure 1C). Additionally, Western blotting analysis confirmed the presence of PD-1 and SIRPα proteins in the hybrid nanovesicles (Figure 1D).

### 3.2 Bioactivity analysis of PD-1/SIRPα NVs

**FIGURE 2:**
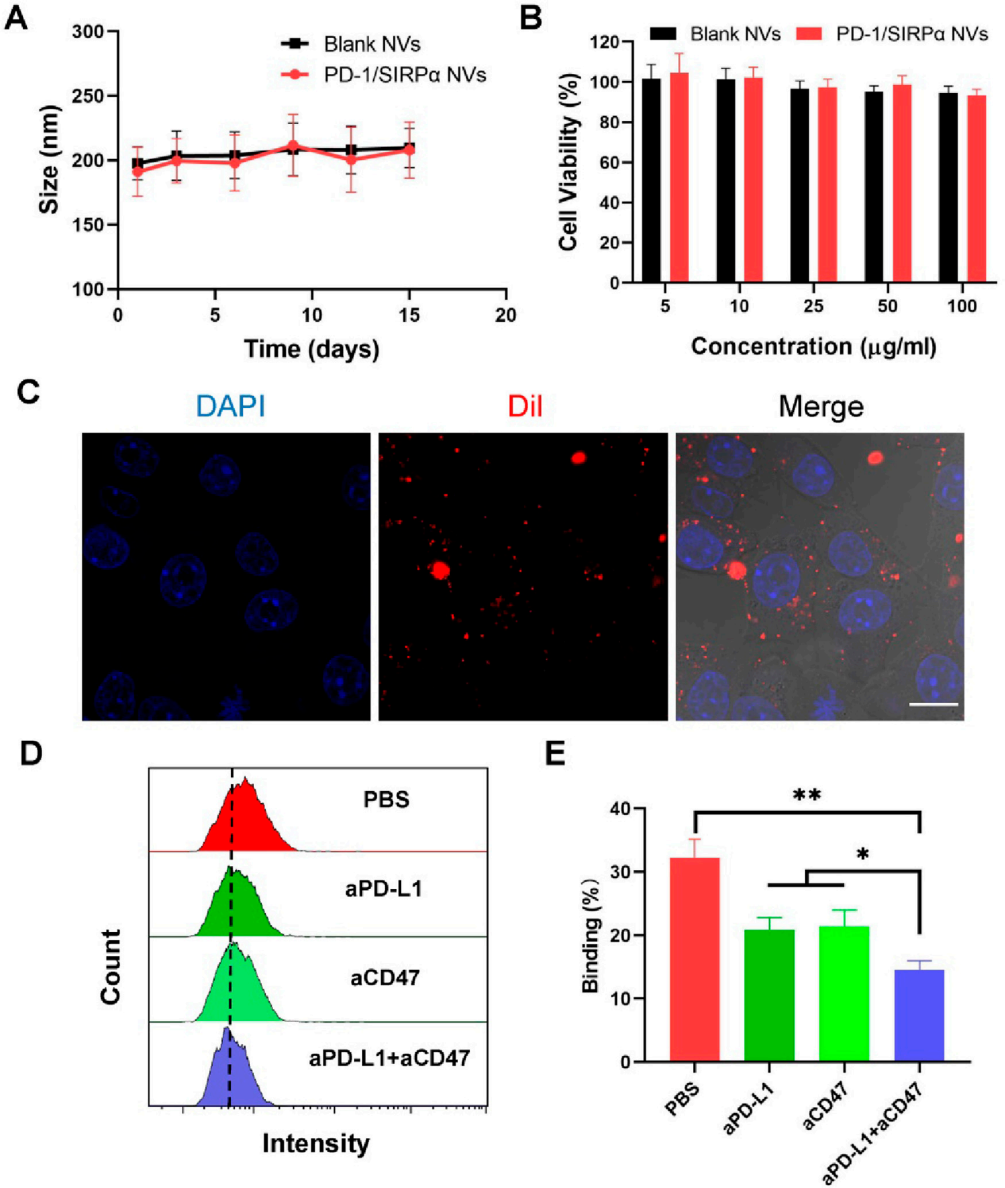
*In vitro* bioactivity analysis of PD-1/SIRPα NVs. **(A)** Stability analysis of PD-1/SIRPα NVs in PBS solution after different days of incubation. The nanovesicles were incubated in PBS with constant shaking at 100 rpm at 37°C. **(B)** Biocompatibility analysis of hybrid nanovesicles on DC2.4 cells by measuring the cell viability of treated cells. DC2.4 cells were treated with Blank NVs or PD-1/SIRPα NVs for 48 h at 37°C. The cell viability of treated cells was analyzed via CCK8 assay. **(C)** Cellular uptake of PD-1/SIRPα NVs in melanoma cells. B16F10 cells were incubated with Dil-labeled PD-1/SIRPα NVs for 2 hours at 37°C. Scale bar: 10 μm. **(D, E)** Flow cytometry curves and quantitative analysis of the blockade property of PD-1/SIRPα NVs. Melanoma cells were pretreated with PBS, anti-PD-L1 antibodies (aPD-L1), anti-CD47 antibodies (aCD47), or a combination of aPD-L1 and aCD47 for 4 hours at 37°C. After washing with PBS, Dil-labeled PD-1/SIRPα NVs were added to each well for a 2-hour incubation. Treated cells were digested and subjected to flow cytometry analysis (gated on PE).

We conducted stability assessments of PD-1/SIRPα NVs in a PBS solution (pH 7.4) over 15 days, revealing no significant changes in size, indicating robust colloidal stability (Figure 2A). Additionally, we evaluated the biocompatibility of PD-1/SIRPα NVs in DC2.4 cells through a CCK8 assay, demonstrating good compatibility across all tested concentrations (Figure 2B). Furthermore, the internalization of PD-1/SIRPα NVs was examined in B16F10 melanoma cells using CLSM analysis. Dil-labeled hybrid nanovesicles were incubated with melanoma cells for 4 hours at 37°C, revealing red fluorescent spots distributed within the cytoplasm, indicating efficient cellular uptake of our nanovesicles in melanoma cells (Figure 2C).

We examined whether our hybrid nanovesicles could block PD-L1 and CD47 ligands on cancer cells simultaneously, given their co-expression of PD-1 and SIRPα receptors. B16F10 melanoma cells, known for PD-L1 and CD47 overexpression, were used for checkpoint block assay. The cancer cells were pre-treated with aPD-L1 and aCD47 antibodies for 4 hours, followed by a two-hour incubation with Dil-labeled PD-1/SIRPα NVs. The resulting cancer cells with fluorescent nanovesicle binding were analyzed using flow cytometry. Our findings showed that aPD-L1 or aCD47 treatments significantly reduced nanovesicle binding compared to PBS treatment. aPD-L1 plus aCD47 treatment resulted in the depletion of approximately half of the tumor-bound nanovesicles (Figures 2D,E). These outcomes underscore the promising potential of PD-1/SIRPα NVs for concurrent immune checkpoint blockade.

### 3.3 PD-1/SIRPα NVs promoted the phagocytosis of melanoma cells by BMDMs

**FIGURE 3:**
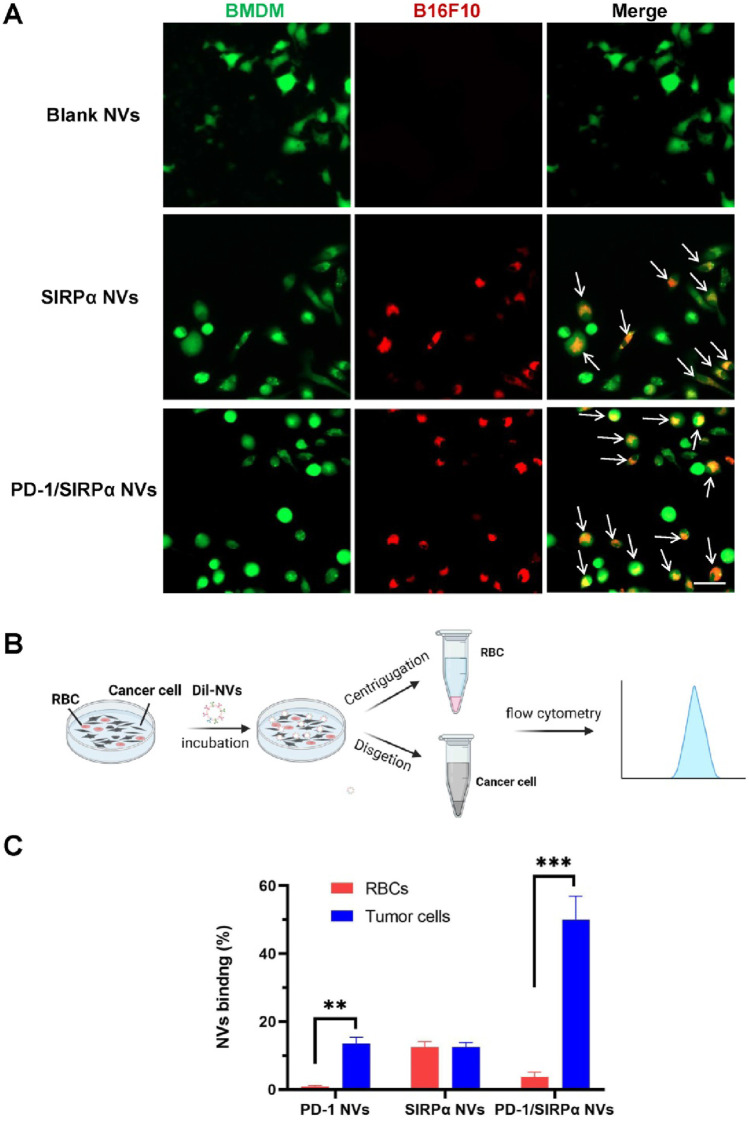
Pro-phagocytosis analysis and selective binding property of PD-1/SIRPα NVs *in vitro*. **(A)** PD-1/SIRPα NVs promoted the phagocytosis of melanoma cells by BMDMs *in vitro*. BMDMs were labeled with CellTracker Green, while B16F10 melanoma cells were stained with CellTracker Red. The phagocytosed cancer cells by macrophages were indicated as yellow (arrow). Scale bar: 20 μm. **(B)** Schematic illustration of selective binding of PD-1/SIRPα NVs with melanoma cells by flow cytometry analysis. **(C)** Quantitive analysis of nanovesicle binding with melanoma cells and red blood cells (RBC) in flow cytometry analysis.

The CD47/SIRPα interaction between cancer cells and macrophages serves as a crucial “don’t eat me” signal, preventing cancer cells from phagocytosis by macrophages (Wang et al., 2022; Zhou et al., 2024). Targeting CD47 blockade can enhance macrophage-mediated phagocytosis of cancer cells, presenting a promising avenue for cancer immunotherapy (Luo J. Q. et al., 2023). In our study, we assessed the pro-phagocytic capacity of PD-1/SIRPα nanovesicles (NVs) *in vitro* due to the presence of the SIRPα receptor. Mouse bone marrow-derived macrophages were cultured and co-incubated with B16F10 cells. Interestingly, in the PBS control group, no phagocytic B16F10 cells (labeled in red) were observed within the BMDMs (labeled in green). In contrast, the treatment with SIRPα NVs or PD-1/SIRPα NVs resulted in the formation of multiple merged yellow spots (indicated by arrows) within the BMDMs (green) (Figure 3A). These findings suggest that SIRPα NVs and PD-1/SIRPα NVs effectively disrupt the CD47/SIRPα axis, significantly enhancing the phagocytosis of B16F10 cells by BMDMs.

The clinical findings indicated that some cancer patients treated with anti-CD47 experienced anemia symptoms (Chen et al., 2022), due to the CD47 expression in red blood cells (RBCs). To explore the tumor-binding property of PD-1/SIRPα NVs, we added Dil-labeled nanovesicles to a dish containing the B16F10 cells and RBCs. After 4 hours of incubation, the suspended RBC and adherent melanoma cells were collected and subjected to flow cytometry analysis (Figure 3B). The results revealed that PD-1 NVs exhibited selective binding with melanoma cells, possibly due to the high expression of PD-L1 on B16F10 cells. Conversely, SIRPα NVs showed no preference in binding between B16F10 cells and RBCs, likely because of CD47 expression on both cell types (Figure 3C). Remarkably, PD-1/SIRPα NVs maintained their tumor-binding preference even in the presence of RBCs. These findings highlight the potential of our hybrid nanovesicles with selective tumor-binding in minimizing side effects such as anemia.

### 3.4 PD-1/SIRPα NVs suppressed tumor growth in mice bearing B16F10 melanoma xenograft

**FIGURE 4:**
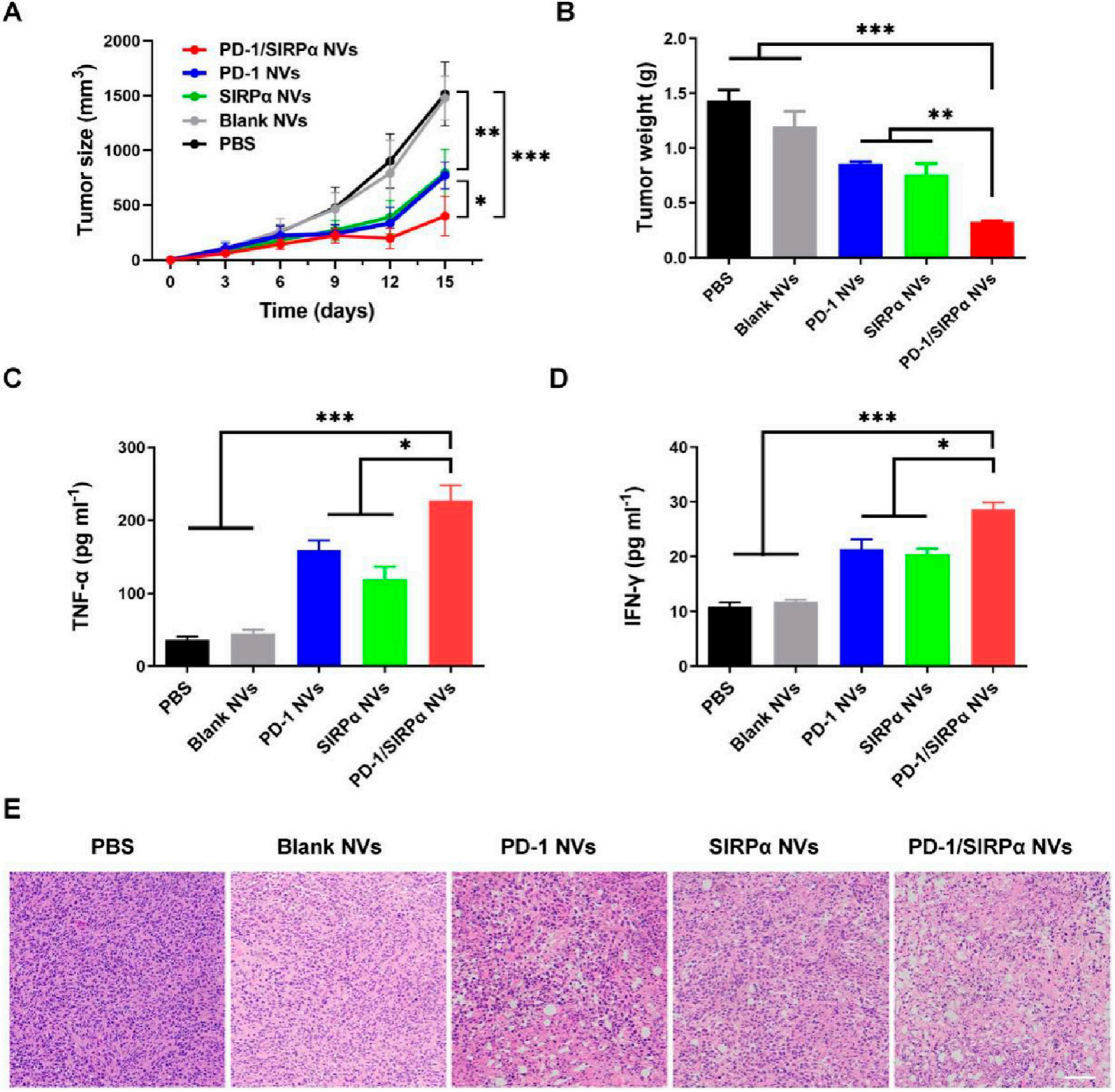
*In vivo* antitumor analysis of PD-1/SIRPα NVs in mice bearing melanoma xenograft **(A)** Tumor growth curves (n = 5). The mice were treated with PBS, Blank NVs, PD-1 NVs, SIRPα NVs, and PD-1/SIRPα NVs five times every 3 days via tail vein injection. **(B)** Tumor weight analysis in different groups. **(C, D)** Serum TNF-α and IFN-γ analysis in mice treated with different formulations. Mouse serum samples were isolated on day 15th after drug injection. **(E)** H&E staining of tumor tissues in different groups. Scale bar: 100 µm. Data were shown as mean ± SD. P values are from one-way ANOVA followed by Tukey’s post-test. *p < 0.05, **p < 0.01, ***p < 0.001.

We further assessed the antitumor efficacy of PD-1/SIRPα NVs *in vivo*. Mice bearing B16F10 melanoma xenografts were established and subjected to treatment with PBS, Blank NVs, PD-1 NVs, SIRPα NVs, and PD-1/SIRPα NVs for five administrations. Notably, PD-1 NVs, SIRPα NVs, and PD-1/SIRPα NVs significantly inhibited tumor growth by day 15, with hybrid nanovesicles exhibiting the most robust tumor suppression effect (Figure 4A). Treatment with PD-1/SIRPα NVs led to a significant 77.2% reduction in tumor weight compared to the PBS group (Figure 4B). Furthermore, we assessed the levels of pro-inflammatory factors in mouse serum and observed a substantial increase in TNFα (Figure 4C) and IFN-γ (Figure 4D) upon treatment with our hybrid nanovesicles, indicative of an induced immune response. Histological examination of tumor tissues through H&E staining revealed evident tumor cell death in mice treated with PD-1/SIRPα NVs (Figure 4E), underscoring a potent therapeutic effect.

### 3.5 PD-1/SIRPα NVs elicited a robust antitumor immune response for effective immunotherapy

**FIGURE 5:**
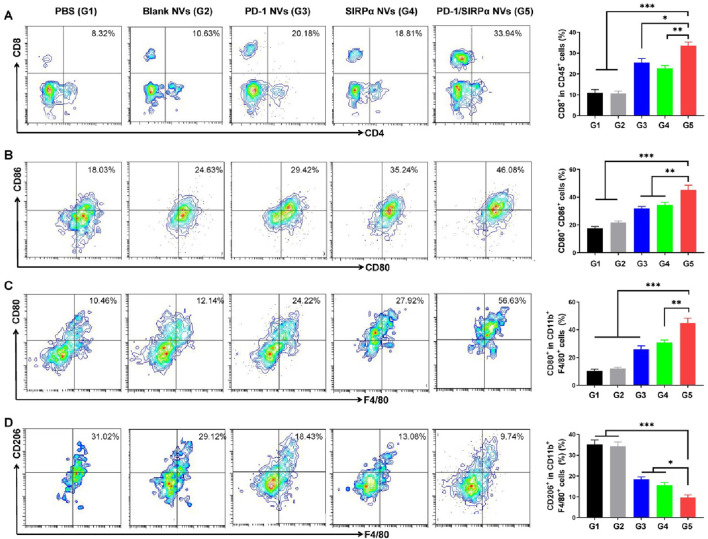
PD-1/SIRPα NVs elicited a solid antitumor immune response in mice bearing melanoma xenografts. **(A)** Flow cytometry analysis and percentage of CD8^+^ T cell infiltration in tumors (gated on CD45^+^CD8^+^ cells). **(B)** Flow cytometry analysis and percentage of mature dendritic cells (gated on CD80^+^CD86^+^ cells). **(C)** Flow cytometry analysis and percentage of M1-type TAM cells (gated on F4/80^+^CD80^+^ cells). **(D)** Flow cytometry analysis and percentage of M2-type TAM cells (gated on F4/80^+^CD206^+^ cells). P values are from one-way ANOVA followed by Tukey’s post-test. *p < 0.05, **p < 0.01, ***p < 0.001.

Given the notable tumor growth suppression effect, we evaluated the tumor-infiltrating immune cells to assess the immunotherapy efficacy. As illustrated in Figure 5A, treatment with PD-1/SIRPα NVs led to a 3.2-fold increase in CD8^+^ T cell infiltration compared to the PBS group. Flow cytometry analysis indicated that our hybrid nanovesicles substantially facilitated the maturation of dendritic cells (Figure 5B). Furthermore, we examined the macrophage polarization induced by PD-L1 and CD47 blockade. Noteworthy is that the results demonstrated a significant enhancement in the percentage of CD80^+^ M1 macrophages (Figure 5C) and a reduction in CD206^+^ M2 macrophages (Figure 5D) upon treatment with PD-1/SIRPα NVs, indicating a polarization towards M1 macrophages. Collectively, these findings underscore the ability of our hybrid nanovesicle to evoke a robust antitumor immune response, showcasing its potential for effective immunotherapy.

**FIGURE 6:**
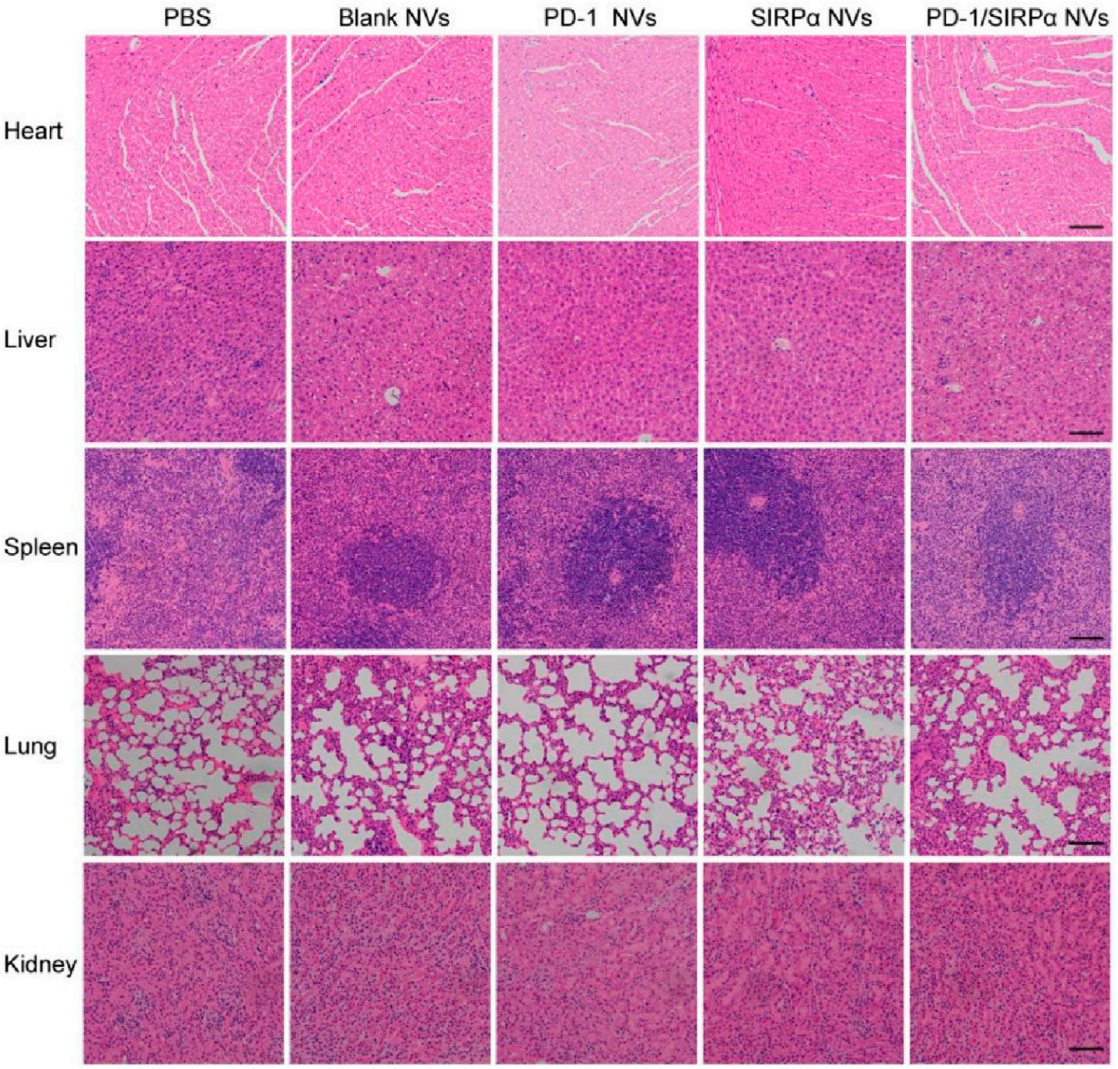
Biosafety analysis of PD-1/SIRPα NVs *in vivo*. H&E staining of main organs including heart, liver, spleen, lung, and kidney from the mice treated with PBS, Blank NVs, PD-1 NVs, SIRPα NVs, and PD-1/SIRPα NVs. Scale bar: 200 μm.

We finally determined the biosafety of our hybrid nanovesicles via pathology examination. At the end of treatment, heart, liver, spleen, lung, and kidney tissues were excised and transformed into frozen slices. Hematoxylin and eosin (H&E) staining was performed to evaluate the side effects of PD-1/SIRPα NVs on normal tissues. The results demonstrated no obvious tissue damage during the treatment (Figure 6), suggesting good biosafety *in vivo*.

## 4 Discussion

ICIs can disrupt the inhibitory signals released by immune checkpoint receptors and restore the immune attack against cancer cells. Given the different roles of immune checkpoints (CTLA-4, PD-1, TIM-3, LAG-3, TIGIT) in immune regulation (de Miguel and Calvo, 2020), concurrent blockade of two immune checkpoints can elicit a high level of antitumor immune response, eventually improving clinical benefit over monotherapy. To this end, some fusion proteins or bispecific antibodies were developed for treating advanced solid tumors (Burton and Tawbi, 2021; Gao et al., 2023). Liu B developed a CD47/PD-L1 fusion protein to trigger innate and adaptive immune responses. Dual blockade of CD47 and PD-L1 by fusion protein induced synergistic antitumor immune response and exhibited potent antitumor activity in the MC38 mouse model (Liu et al., 2018). Wang R designed a CD47/PD-L1 bispecific antibody (6MW3211) for enhanced immunotherapy. 6MW3211 efficiently disrupted PD-1/DP-L1 and CD47/SIRPα signaling and demonstrated potent therapeutic effects in three tumor models (Le et al., 2021). Ma L et al. fabricated a PD-L1/TIGIT bispecific nanobody to disrupt PD-1/PD-L1 and TIGIT/CD155 interaction. The bispecific nanobody synergistically enhanced T cell function (Ma et al., 2020).

Cell membrane nanovesicles, derived from natural cell membranes, offer numerous advantages as a drug delivery platform for cancer therapy, including reduced clearance by the reticuloendothelial system and prolonged circulation time. Notably, engineered cell membrane nanovesicles can display membrane receptors on their surface, allowing them to perform the intrinsic functions of these receptors. In this study, we decorated cell membrane nanovesicles with PD-1 and SIRPα receptors to enhance immunotherapy efficacy in melanoma. The resulting hybrid nanovesicles demonstrated high specificity with PD-L1 and CD47 ligands expressed on melanoma cells. Notably, our hybrid nanovesicles exhibited mild binding with red blood cells (RBCs) compared to their strong interaction with melanoma cells. This difference in binding affinity can be attributed to the presence of only CD47 on RBCs *versus* the co-expression of PD-L1 and CD47 on melanoma cells. This suggests that PD-1/SIRPα nanovesicles may pose a low risk of blood-related side effects, such as anemia. However, the long-term safety profile and potential off-target effects of PD-1/SIRPα nanovesicles need to be investigated in future research.

The blockade of CD47 by our nanovesicles significantly enhanced the phagocytosis of melanoma cells by macrophages, facilitating the release and presentation of tumor-associated antigens and thereby promoting the maturation of dendritic cells. Additionally, disrupting the PD-L1/PD-1 axis with our nanovesicles reinvigorated exhausted cytotoxic T cells, eliciting a robust immune response against tumor cells. Consequently, in mice bearing melanoma xenografts, PD-1/SIRPα nanovesicles substantially promoted the infiltration of CD8^+^ T cells and the polarization of M1-type tumor-associated macrophages (TAMs), collectively enhancing therapeutic outcomes. These findings suggest that cell membrane nanovesicles decorated with two or more checkpoint receptors hold promising potential for treating cancers and immune-related diseases. To fulfill the therapeutic potential of PD-1/SIRPα nanovesicles, future studies should be performed to optimize the hybrid nanovesicles. Several factors, including suitable cells for gene transfection, the optimal size of nanovesicles, receptor protein concentration, and the molar ratio of the two receptors, will determine the therapeutic outcomes of nanovesicles.

## 5 Conclusion

In summary, we developed a hybrid cell membrane nanovesicle for concurrent immune checkpoint blockade in melanoma. Our hybrid nanovesicles can promote the efficient phagocytosis of melanoma cells by macrophages. PD-1/SIRPα NVs significantly suppressed tumor growth and elicited a robust antitumor immune response with enhanced immunotherapy efficacy. In conclusion, our findings highlight the promising potential of PD-1/SIRPα NVs as novel therapeutic agents for cancer immunotherapy.

## Data Availability

The original contributions presented in the study are included in the article/supplementary material, further inquiries can be directed to the corresponding author/s.
